# Abstract of Proceedings of the Buffalo Medical Association

**Published:** 1866-07

**Authors:** T. M. Johnson

**Affiliations:** Sec’y.


### ART. V.—*Abstract of Proceedings of the Buffalo Medical Association.*

TUESDAY EVENING, June 5th, 1866.

The President, Dr. Gould, in the Chair.

Present—Drs. Gould, Gay, Cronyn, Whitney, Trowbridge, Rochester, Tafft, Hazen, Smith, Gleason, Ring, Boardman, Greene, Wetmore and Johnson.

The reading of the minutes of the last meeting was, on vote of the Association, dispensed with.

DR. ROCHESTER related the following case of puerperal eclampsia: Was called a few weeks ago to the case, but being engaged at the time sent my associate, Dr. Abbott, who found the patient in puerperal convulsions, and advanced to the eighth month in pregnancy. Dr. Abbott immediately gave chloroform to anæsthesia. Called myself an hour after and found the patient with a full, strong pulse, rather plethoric and severe headache. Bled freely from the arm. Soon after the arm was tied up another convulsion came on. Gave chloroform to anæsthesia. Two hours later another convulsion came on; after which I took about the same quantity of blood from the arm as at the first bleeding, having taken in all about thirty-two ounces of blood; after which she had no more convulsions After two days the labor came on when she was delivered by forceps.

I believe that chloroform alone is insufficient to control the convulsions in a majority of these cases. Blood-letting and chloroform combined are much more efficient. Blood-letting is as useful now as ever it was, and should be resorted to in cases like the one just related. Would call the attention of the Association to an article upon this subject in the *Buffalo Medical Journal*, written by Dr. Newman.

DR. GOULD mentioned a case similar to the one just related by Dr. Rochester, in which blood-letting and chloroform were combined, with excellent results.

DR. CRONYN remarked that he regretted that the treatment of such cases by blood-letting had been so much ignored within the last few years. He believes that in plethoric cases, with full, strong pulse, the blood-letting quite as useful as the chloroform. In hysterical cases chloroform alone is very nseful, and usually will control the convulsions. In apoplectic or epileptic cases bloodletting is very important and useful. Keeping the patient under the influence of chloroform for a proper length of time, after blood-letting, is very useful.

Dr. Gay reported the following case:

A. B. N., aged 39 years, of intemperate habits, drank to excess for two days, when he became very violent, and was sent to Buffalo General Hospital. When called to see the patient found him almost lifeless, the surface presenting a very livid appearance, the respiration very labored, had in fact almost ceased; deglutition impossible; pulse would cease for considerable time and then returned feebly. Opened the jugular vein, when the blood was at first thick, almost coagulated; at the expiration of about five minutes the blood flowed freely until about thirty-two ounces had been taken, when the opening was closed. After the bleeding ceased the pulse became fuller and stronger and quite regular. Respiration improved, and he became conscious a few hours after. Two days later his left arm became red and swollen, and on the eleventh day he died of erysipelas. The patient's life was undoubtedly prolonged the eleven days by the means employed by Dr. Shirley and the superintendent of the hospital.

Dr. Gay stated that the portrait of Dr. Josiah Trowbridge was in Mr. Hill's studio, and requested the Secretary to have it placed in the rooms of the Association.

Adjourned. T. M. Johnson, Sec'y.

